# Nutrient Density, Added Sugar, and Fiber Content of Commercially Available Fruit Snacks in the United States from 2017 to 2022

**DOI:** 10.3390/nu16020292

**Published:** 2024-01-18

**Authors:** Hao Fu, Chi Hyun Lee, Alissa A. Nolden, Amanda J. Kinchla

**Affiliations:** 1 Department of Food Science, College of Natural Science, University of Massachusetts, Amherst, MA 01003, USAanolden@umass.edu (A.A.N.); 2 Department of Biostatistics, School of Public Health, University of Massachusetts, Amherst, MA 01003, USA

**Keywords:** nutrition profile, energy density, nutrient rich food index, nutrition quality

## Abstract

Fruit snacks have become a popular and convenient snacking choice and have the potential to contribute to a well-balanced diet. However, the nutritional quality of fruit snack products has not yet been studied. The objective of the present study is to provide a nutritional assessment of the fruit snack product category. This study used the Mintel Global New Product Database to collect data about fruit snack products launched in the United States from 2017 to 2022. Fruit snack products (*n* = 2405) are divided into nine product categories based on product characteristics. Nutrition composition was assessed using a comprehensive score, Nutrient Rich Food (NRF) model, and by examining individual components (added sugar and fiber). The results show that dried fruit has the highest nutrient density, fiber content, and the lowest added sugar content. Conversely, fruit-flavored snacks have the lowest nutrient density, fiber content, and added sugar content. Currently, fruit puree, canned fruit with juice, and dried fruit are the only fruit snacks that meet the current recommendations set by the USDA Dietary Guidelines. Future directions for the fruit snack category should consider decreasing the added sugar content, increasing the fiber content, and enhancing their sensory profile to improve the overall nutrient density.

## 1. Introduction

Fruit is one of the most frequent snacking recommendations made by many organizations across the globe [1]. The 2020–2025 USDA Dietary Guidelines recommend incorporating fruit into the diet since it is nutrient-dense and provides a good source of fiber; however, 80% of the American population does not meet the daily amount of fruit servings recommended by Dietary Guidelines for Americans [1,2]. To help increase the number of servings of fruit in their diet, consumers can choose nutrient-dense snacks as one strategy to address the gap in their fruit consumption [1,2]. Fruit-based snacks, like dried fruit and fruit bars, provide a convenient vehicle to deliver health-promoting components, including vitamins and antioxidants [3]. According to the National Consumer Survey (2015–2016), 26.5 million households in the United States consumed fruit snacks, and 23% of these households consumed eight or more snacks in a month [4]. Reports over the past 40 years indicate a surge in snacking between meals. Within this period, cross-sectional data from National Health and Nutrition Examination Surveys (NHANES) indicate that the ratio of energy intake from snacks to daily energy intake has increased, whereas the percentage of energy from meals has declined [5].

However, the health effects of snacking are concerning as it has been linked with the etiology of obesity as snacking is estimated to comprise 20–25% of the average consumer’s total daily energy intake, based on the 2013–2016 National Health and Nutrition Examination Survey (NHANES) [6]. Additionally, snack foods are associated with an increased daily consumption of added sugar and contribute to a positive energy balance, which is linked to having a higher BMI [7,8,9]. Although the average added sugar consumption has decreased over the past decade, the percentage of total calories consumed from added sugars exceeds the dietary recommendation (less than 10% of daily energy intake) over the past four decades [10,11].

Nutrition profiling is a quantitative method used to assess the nutrient density of foods based on their nutrient content [12,13]. Several different analytical models have been developed to assess the degree of “healthfulness” of food, which also serves as the scientific basis for food product reformulation in the industry [14]. The Nutrient Rich Foods (NRF) model has been widely used to evaluate the nutrient density of individual foods, composite meals, and the total diet [12,15,16,17]. The NRF model is a comprehensive guidance system used to quantify an individual food’s nutrient density by using numerical values as indicators of the healthfulness of foods. This approach helps to provide a novel approach for comparing nutrient profiles when comparing food choices. The advantage of this approach is the flexibility of the model, as it allows for the selection of different nutrient attributes depending on the specific product category. As new recommendations or policy updates become available, the algorithm structure is maintained, but the nutrients and calculation parameters can be easily added, removed, or updated [18]. Several studies have applied the NRF model to assess the nutrient density of snacks [16,19,20,21]; however, no study has applied the NRF model to assess the healthfulness of fruit snacks.

With the most recent changes to the United States Nutrition Facts panel, the Daily Value of nutrients, which are the parameters in NRF nutrition profiling algorithms, have recently changed, as the FDA issued updates to the Nutrition Facts label for the first time in over 20 years, including added sugar, vitamin D, calcium, potassium, and updated the values for percent Daily Value (%DV) [22]. These changes took effect in July 2016 after first being proposed in May 2016, and were required to be fulfilled no later than 2021. Therefore, the NRF model for the fruit snack category can help us to assess the nutrient density of foods using the updated Nutrition Facts Label. For example, the Dietary Guidelines for Americans updated their recommendations to limit the number of calories from added sugars to be less than ten percent of total calories per day [23]. As such, the revised labeling requires foods to declare added sugars on the facts panel, as many consumers are unaware of where or how much sugar can be in foods. Due to the flexibility of the NRF model, added sugar can be added to the formula.

While the total snack category contributes to a high percentage of added sugar and total energy intake, there is a gap in the literature regarding fruit snacks’ nutritional composition and degree of healthfulness. In this study, we aim to address this knowledge gap by performing a nutrition profiling assessment of the nutrient density of fruit snacks by using the optimized NRF model and comparing the added sugar and dietary fiber content of all fruit snack products on the market. This will contribute to a new understanding of the healthfulness of products in the fruit snack category and may be used to improve the nutritional composition of fruit snack products in the future.

## 2. Materials and Methods

### 2.1. Overall Approach

The Mintel Global New Product Database (GNPD) platform is utilized to source information about fruit snacks products available on the market. Fruit snack products are further classified into 9 subcategories based on the fruit snack product category and the research scope. Product categories were analyzed for their added sugar, fiber content, and energy (calories), and the NRF score was calculated.

### 2.2. Definition of Fruit Snacks

The definition of snacks, including snacking and snack foods, is controversial, as there is a lack of a consistent definition of the term [24,25]. The USDA Commercial Item Description describes fruit snacks as “products made with fruit and fruit juices, which may or may not contain added sugar, artificial colors and flavors, and preservatives” [26]. For this study, “fruit snacks” are defined as non-frozen, non-beverage food products mainly made with fruit ingredients, including products such as fruit leather, fruit rolls, fruit-based bar, dried fruit, fruit gummies, and canned fruit. Fresh or unprocessed fruit were excluded from the scope.

### 2.3. Search Criteria

A systematic search was carried out using the Global New Product Database (GNPD) platform [27], launched by MINTEL, to generate a database of fruit snack products commercially available in the U.S. Fruit snack products were collected by applying multiple search criteria (see key words below). The search query included a five-year period of products available in the U.S. market from 1 January 2017 to 31 December 2022. This time frame was selected due to FDA’s new regulation on food product nutrition labels, which went into effect on 26 July 2016. The search was performed within two existing categories available in GNPD product category options, “Fruit snacks” and “Fruits”. An additional search was performed to collect products which qualified as fruit snacks but were not categorized in the “Fruit snacks” category in the GNPD by using the keyword “fruit” in the following categories: “Sugar & Gum Confectionery”, “Shelf-Stable Dessert”, and “Snack Bar”. Products could be either chilled or shelf stable.

Product information exported from MINTEL included the fruit snacks’ record ID, brand name, product name, product image, date launched, product description, storage type, package type, product claims, ingredients list, serving size, serving measure (g), energy (kcal), saturated fat (g), trans fat (g), cholesterol (g), sugar (g), fiber (g), protein (g), potassium (mg), vitamin D (mg), calcium (mg) and iron (mg), and percentage of the daily value for each nutrient.

### 2.4. Data Management

Data cleaning was performed using Pandas Package [28] in Python 3.7.4 [29]. Products were removed if they did not meet the search criteria for fruit snacks or did not have nutrition labels. Products were screened for overlapping entries and duplicate products were removed. Any fruit snack products that had missing or incomplete nutrient data were cross-referenced on the official product website and large retailer websites to obtain missing nutrient content information. For products that have dual or multiple labels, an average of nutrient contents was calculated to represent the nutrient content of the product. Any products that contained missing values for added sugar, vitamin D, calcium, or iron were removed from the dataset.

Fiber content data noted as “<1” were replaced by the estimated numerical data, calculated based on the daily recommendation value and daily value (%DA) indicated on the nutrition label. Saturated fat, sodium, and cholesterol content recorded as <0.1 g or <5 mg was replaced by 0.1 g and 5 mg, respectively. Protein content recorded as <1 g was entered as 0.75 g. The serving size of all fruit snacks was aligned to grams (g).

Based on the product descriptions provided in the final database, products were placed into subcategories. There are various types of commercially available fruit snacks on the U.S. market, such as dry fruit, fruit cup, fruit gummy, fruit bar, fruit leather, fruit roll, etc. Therefore, fruit snacks were further classified into nine different fruit snack categories based on the fruit snack products’ standard form and commercial popularity: dry fruit, fruit-based bar, dry flavored fruit, canned fruit, fruit-flavored snack, fruit puree, fruit chips, formed fruit, canned fruit with juice. A description of each fruit snack subcategory is provided in Table 1.

### 2.5. Nutrient Profiling

The algorithm for the Nutrient Rich Food (NRF) model comprises two parts, nutrient rich, “NR” (sub scores of nutrients to encourage) and nutrients to limit, “LIM” (sub scores of nutrients to limit). NR is a selection of nutrients that are essential for health, like protein, fiber, vitamins, and minerals recommended by the FDA, which are also referred to as qualifying nutrients. LIM are nutrients which are detrimental to health when intake is excessive and should be limited, which is called disqualifying nutrients, this includes saturated fat, sodium, added sugar, and cholesterol [12,13,30]. The NRF*_n.m_* score was calculated by Drewnowski and Fulgoni (2020) and is shown in Equation (1) [31].

(1) NRFn.m=NRn−LIMm 

In Equation (1), *n* represents the number of nutrients to encourage, and *m* represents the number of nutrients to limit. In published studies, the number of nutrients selected for *LIM* (nutrients to limit) is usually 3, but the number of qualifying nutrients (*NR*) that should be encouraged has varied from 6 (NRF6.3) to 15 (NRF15.3) [13,19,20,32].

In this study, we used the NRF6.4 model (*n* = 6 and *m* = 4) to assess fruit snack nutrient density. All nutrient daily values are based on the FDA’s updated Nutrition Facts label requirements for packaged foods and drinks in 2016 [22]. As mentioned above, added sugars are now declared on the label to provide additional information to help consumers make informed decisions. Vitamin A and C are no longer required to be on the label, whereas Vitamin D and Potassium are now required to be listed on the label as the average American consumes below the recommended amounts [22]. The Daily Values used on nutrition labels and published by the FDA are summarized in Table 2.

The percentage daily values for nutrients were capped at 100%. The NRF6.4 model for fruit snacks was calculated as seen in Equation (2).

(2) $$\mathrm{NRF}\;6.4=\sum_{i=1}^{6}\frac{Content\;i}{DVi}\times 100-\sum_{j=1}^{4}\frac{Content\;j}{DVj}\times 100\;$$

which is based on the updated nutritional facts and qualifying nutrients for *NR* (Protein, Dietary Fiber, Potassium, Vitamin D, Calcium, and Iron) and *LIM* (Saturated Fat, Cholesterol, Added Sugar, and Sodium). Where *i* = qualifying nutrients in *NR*, *j* = disqualifying nutrients in *LIM*, and the basis of calculation was 100 kcal, as used previously [18,19,20]. The energy density was calculated by converting calories per serving on the package to calories per 100 g as additional information for nutrient profiling.

### 2.6. Added Sugar and Fiber Content in Fruit Snacks

Nutrient values are reported per serving size. Added sugar and fiber per 100 g were calculated to account for the variability in serving sizes across fruit snack products. Additionally, added sugar and fiber content were calculated based on the FDA’s Reference Amount Customarily Consumed (RACC) per eating occasion to balance the serving variability among different fruit snack categories. RACC values are set at 40 g for dried fruit, flavored dried fruit, and fruit-based bar; 30 g for formed fruit, fruit-flavored snack, and fruit chips; 140 g for frozen fruit, fresh fruit, and canned fruit; and 125 g for fruit puree [33].

### 2.7. Statistical Analysis

We summarized nutrition profiling using the mean and standard deviation of the NRF score. For added sugar and fiber content, the mean and standard deviation were reported. Since the NRF score, added sugar content, and fiber content data are not normally distributed, non-parametric methods—Kruskal–Wallis testing followed by Dunn post hoc analysis with holm p-adjustment approach—were used to analyze the differences in NRF score, added sugar content and fiber content among the 9 fruit snack product categories pair-wisely. The statistical analysis was conducted in R, version 4.0 [34] by using packages ‘tidyverse’ and ‘FSA’ version (0.8.32) [35,36].

## 3. Results

After carrying out the search, 2874 fruit snack products were initially identified. Of these products, 362 did not align with the fruit snacks’ product category description, and 107 did not include nutrition labels nor could be found through additional searches and were removed from the study, which resulted in 2405 fruit snacks being included in the final dataset for the analysis of fruit snack product launch.

Due to the ongoing transition to the new Nutrition Facts label, which impacted requirements for reporting added sugar, Vitamin D, Calcium, and Iron on the packaging, products were further screened for reporting these nutrients. Fruit snacks with missing nutrient data were cross-referenced with the official product website and large retailer websites. A total of 908 products were missing these nutrients and were thus removed from the database. This resulted in a total of 1497 fruit snacks being included in the dataset for fruit snack product nutrition profiling and nutrient content analysis.

### 3.1. Fruit Snacks Launched to U.S. Market from 2017 to 2022

The number of fruit snacks launched in the U.S. Market during 2017–2022 is summarized according to fruit snack category (see Table 3). Notably, dried fruit, fruit-based bar, and dried flavored fruit rank as the top three leading product categories launched to market in the fruit snack category.

### 3.2. Nutrition Profiling of Fruit Snacks

Here, we report the NFF6.4 score calculated for each product category (see Table 4). The average NRF6.4 score ranges from −29.5 (fruit-flavored snack) to 20.9 (dried fruit). The average energy density ranges from 72.2 kcal/100 g (canned fruit with juice) to 509.5 kcal/100 g (fruit chips).

The Kruskal–Wallis test revealed significant differences in NRF6.4 score among the 9 fruit snack product categories (df = 8, *p* < 0.0001). Canned fruit with juice and fruit puree have a low energy density, even though they have a high NRF 6.4 score.

**Figure 1:**
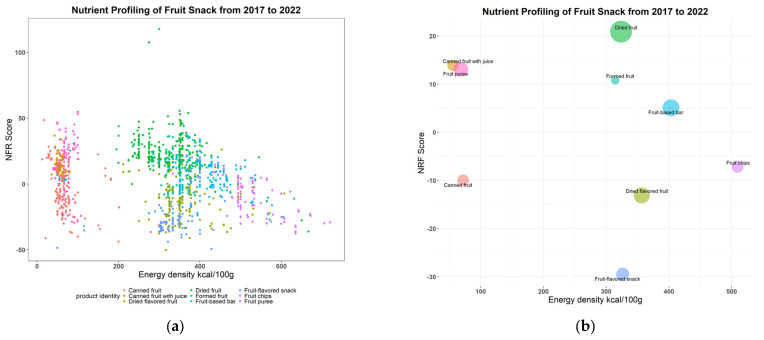
(**a**) NRF6.4 score and energy density for each fruit snack product in the dataset). (**b**) Mean NRF6.4 score of each fruit snack product category plotted again mean energy density (kcal/100 g), the size of the bubble denotes the number of fruit snacks in each product category.

As shown in Figure 1, NRF6.4 score is plotted against energy density (kcal per 100 g). Figure 1a is a scatterplot of the relation between energy density of individual fruit snack products and NRF6.4 score. Mean NRF6.4 scores in relation to the mean energy density of each fruit snack product categories are mapped in Figure 1b. The size of the circle indicates the relative proportion of products in each category. Canned fruit with juice has the lowest energy density (insert mean and std deviation 55.9 ± 7.6) and the second highest NRF6.4 score (13.9 ± 6.8). Even though dried fruit and fruit-flavored snacks have a similar energy density (323.8 ± 62.4 and 326.2 ± 45.9, respectfully), dried fruit has the highest NRF6.4 score (20.9 ± 12.5), whereas fruit-flavored snacks have the lowest NRF6.4 score (−29.5 ± 6.4).

### 3.3. Added Sugar and Fiber Nutrient Content

The mean energy density, added sugar content, and fiber content, together with standard deviation per RACC and per 100 g, are displayed in Table 5. Kruskal–Wallis testing showed significant differences among the nine different fruit snack product categories for added sugar per RACC (df = 8, *p* < 0.0001), added sugar per 100 g (df = 8, *p* < 0.0001), fiber per RACC (df = 8, *p* < 0.0001), and fiber per 100 g (df = 8, *p* < 0.0001).

Fruit-flavored snacks had the highest average added sugar content either per RACC or per 100 g, providing 14.6 ± 3.5 and 48.6 ± 11.7, respectively. In terms of the lowest added sugar content, dried fruit was the lowest based on RACC (0.2 ± 1.6), while canned fruit with juice was the lowest based on per 100 g (0.1 ± 0.8). As for fiber content, dried fruit had the highest average fiber content for both per RACC and per 100 g (3.5 ± 2.1 and 8.8 ± 5.3), while fruit-flavored snacks had the lower fiber content for both per RACC and per 100 g (0.1 ± 0.6 and 0.4 ± 1.9, respectively).

## 4. Discussion

Starting in 2015, the USDA Dietary Guidelines for Americans has recommended choosing snack products with low energy but a high nutrition density, and limiting the number of calories from added sugar to less than 10 percent of daily energy intake [2,37]. Here, the data show that only fruit puree and canned fruit with juice meet the dietary recommendations for low-energy snacks and high nutrition profiles (Table 5 and Figure 1). It should be noted that, despite dried fruit having the highest nutrient density and fiber content and the lowest added sugar content among all the fruit snack product categories, its energy density is the fifth highest per 100 g. However, this is largely due to the calculation of energy density being based on amounts which are higher than typical consumption amounts (e.g., 100 g). In contrast, the RACC is 40 g, and as a result, is the fourth highest energy density, following fruit based-bar, fruit chips, and flavored dried fruit.

This finding highlights the importance of examining multiple nutrient characteristics and not merely relying on energy density alone. In other words, energy density may not provide a complete assessment of the nutritional impact of a food. In the present study, the nutrient density and NFR model allow for a more comprehensive investigation and comparison of the nutrient composition of fruit snacks.

### 4.1. Application of NRF Model in the Fruit Snacks Category

The NRF model used in this study incorporates nine nutrients that it is mandatory to list on a nutrition label and are regulated by the FDA. In previous studies, nutrient type in the NRF model has ranged from nine to eighteen nutrients based on different research scopes or targets [13,16,17,30,38].

A previous study used the NRF9.3 model to assess nutrient density in the fruit category [18]; however, dried fruit is the only product category that overlaps with this study. Interestingly, the NRF scores for dried fruit in these two studies are not similar due to differences in the selection of qualifying nutrients and limiting nutrients. Moreover, the dried fruit snacks category in the present study was classified into dried fruit and dried flavored fruit, based on the addition of flavor.

Fruit is an important source of micronutrients and bioactive phytochemicals in the human diet, such as carotenoids, polyphenols, flavonoids, vitamins, and minerals [39]. However, depending on fruit varieties, parts, and growth stage, their nutrient compositions and contents can be different [40]. Total Flavonoids have been incorporated into the NP model to recalibrate and assess the total nutritional value of the fruit [18]. Therefore, including additional nutrients that pertain to fruit-based products in the NRF model in the future, such as bioactive phytochemicals, vitamins, and minerals, could provide further insights into understanding the nutrient density and content of the fruit snack category.

### 4.2. Fruit Snacks’ Nutrient Density and Nutrient Content in Each Fruit Snack Category

The USDA Dietary Guidelines 2020–2025 indicate that fruit can either be consumed in nutrient-dense forms, like whole fruit, or in processed forms of foods that may not be nutrient dense [2]. Fruit snacks are often perceived as contributing to total fruit consumption and are often considered to be nutritious [41]. However, the present data demonstrate a wide variability in their nutrition profile, specifically in terms of NRF score, energy density, dietary fiber, and added sugar content. This variability is likely driven by the processing method used (drying, canned, puree, etc.), along with the use of different ingredients and formulations across fruit snacks. It is important to note that, even within a fruit snack category, there is wide variability, suggesting that both the processing method used and their ingredients are driving the variability in nutrient composition. Here, we provide a summary of each fruit snack category and briefly consider the potential impact of processing and ingredients on the nutrient composition.

Dried or dehydrated fruit makes up one-third of the fruit snacks analyzed in this study, which is further subdivided into two groups based on the addition of flavor, with 62% containing no flavor (i.e., dried fruit) and 38% containing additional flavor (i.e., dried flavored fruit). Regarding all dried fruits, unflavored dried fruit is more nutrient-dense and contains less added sugar than dried flavored fruit. On average, dried flavored fruits contained slightly more than three teaspoons more added sugar per RACC than unflavored dried fruit. This was expected as drying flavored fruit, usually flavored by ingredients like added sugar, salt, spice, and oil, is commonly used for preserving flavor and preventing spoilage. However, dried fruit and dried flavored fruit are similar in product appearance, and it is not well understood if consumers are aware of the nutritional differences between flavored and unflavored dried fruit.

Canned fruit in juice, on the other hand, is more nutrient-dense than canned juice and contains three fewer teaspoons of added sugar per RACC than canned fruit. The inclusion of fruit juice or liquid in canned fruit contains naturally occurring sugar, but it is not included in the calculation of added sugar. Canned fruit is preserved in a variety of liquids, such as syrup, salt water, or artificial/natural sweetener solutions. The calories from nutritive sweetened liquids increases the overall calories (energy), thereby increasing the energy density.

“Formed fruit” refers to the collection of fruit snack products processed into certain shapes, such as leathers, jerky, rolls, or twists, which have become popular and are perceived as a healthy snack in the U.S. [42,43]. The nutrition density of formed fruit is similar to fruit-based bars, fruit puree, and canned fruit with juice. Formed fruit is processed by drying a thin layer of fruit puree, and the dehydration process involved in formed fruit processing renders the formed fruit sticky, chewy, and soft texture [43,44]. These drying methods remove water and maintain the fiber, minerals, and micronutrients [45]. The average added sugar content in formed fruit is 3.4 g per RACC. One explanation for this is that fruit juice concentrate is often added to formed fruit products.

Fruit puree products, packed in ready-to-eat (RTE) pouch packaging, have gained huge popularity as convenient, complementary foods in recent years [46,47], especially in the infant and toddler food category. Perhaps due to their success, fruit puree pouches are being marketed to adults as a convenient snack [48]. Our data reveal that fruit puree, relative to other fruit snacks, has a high nutrient density, low energy density, and low added sugar and moderate fiber content per RACC. These fruit puree products are made by pureeing pectin-rich whole fruits and adding other fruits to enhance the flavor and texture, reducing the need to add extra sugar or syrup [49].

Fruit-based bars fall under the category of a snack bar. The various components, such as nuts, seeds, cereal, and soluble and insoluble dietary fiber, constitute a compact product to provide energy and provide a source of slowly digestible carbohydrates and micro- and macronutrients [50,51]. Popularity and a continuous increase in snack bar consumption prompts the reformulation of and addition of new ingredients into snack bars [51]. Fruit can be used as an alternative natural substitute for sugar, honey, or syrup, for example, date palm fruits, and can enhance the antioxidant, dietary fiber, mineral, and vitamin content of snack bars [52]. The data in our study show that fruit-based bars have a high energy density and fiber content per RACC. The average amount of added sugar in fruit-based bars is 6.1 g per RACC, which we expect to be driven by added sugars, honey, and syrups used to bind ingredients. In addition, a fruit filling in a fruit-based bar usually contains various types of added sugar, such as sucrose, juice concentrations, and syrup.

Even though fruit chips provide 2.3 g of fiber with only 1.7 g of added sugar per RACC on average, the overall nutrient density of fruit chips is relatively low. This can be explained by the fact that the ingredients and procedures which are in processing fruit-chip, such as oil, sodium, and frying methods, result in saturated fat, cholesterol, and sodium in the final products.

In this study, the fruit-flavored snack represents the group of fruit snack products that are similar to “gummies”, yet the products in this data set contain fruit. Our data reveals that fruit-flavored snacks have the lowest nutrition density and fiber content, and the highest added sugar content per RACC, among all fruit snack product categories. One reason for this is that the primary ingredient is usually fruit juice concentrate, sucrose, or high-fructose corn syrup. Incorporating fruit components into candy formulation could be an opportunity to mitigate health issues resulting from the consumption of common sweets by providing bioactive compounds and natural colorants [53]. However, even though these products contain fruit, others have noted concerns about the health and nutrition level of these products [54,55]. It has been suggested that consumers need more education on understanding packaging labels and reading ingredient lists to increase their awareness of added sugar [55,56].

Past research has demonstrated that, within the snack category, products are higher in nutrients that should be limited in consumers’ daily diet and lower in nutrients needed to meet daily recommendations [19]. Our study demonstrated that this finding also applies to the fruit snack category, even though consumers often perceive that fruit snacks are healthy and can be a good source of fiber [57,58,59,60]. The data in our study suggest that nutrient density, added sugar, and fiber content vary significantly across specific product categories. In addition, the perceived healthiness of food or cognitive factors, such as common beliefs, type of food, and branding, can contribute to a judgment on the impact of the perceived healthiness [61]. However, nutritional information can counteract this bias and facilitate rational food choices [61]. Therefore, future research could investigate the association between product claims and nutrition density among different product categories in the fruit snack category.

### 4.3. Recommendations and Strategies for Improving the Fruit Snack Nutrient Composition

The reformulation of fruit snacks is needed, as dried fruit and fruit puree are the only two fruit snack categories which are nutrient-dense, high in dietary fiber, and low in added sugar, and meet the recommendations in the USDA Dietary Guidelines [2,37]. Formed fruit and fruit-based bars could be lower in added sugar to become a more nutritious fruit snack option. Canned fruit and fruit-flavored snacks need more reformulation, as they are low in nutrient density and fiber content and high in added sugar. Improving the nutritional quality of fruit snacks can facilitate smart snacking choices.

The sensory properties of food, aroma, appearance, flavor, and texture can be captured by independent sensory systems and integrated into an overall perceptual impression, which impacts humans’ intake behavior and energy selection [62]. Evidence shows that harder and chunkier solid foods result in less food consumption [63]. A high palatability and food-related ambient odors can increase a particular food choice among other food options, which are normally associated with liking and positive emotions [64]. Liem and Russel [65] have shown that, typically, nutrient-poor food products attract consumers via their sensory profile, in that nutrient-poor foods are likely to be sweet, salty, and have a fatty mouthfeel, while the sensory profile of nutrient-rich foods is diverse. Therefore, developing or improving the sensory profile of nutrient-dense fruit snacks, such as fruit puree, canned fruit with juice, and dry fruit, is a way to encourage healthier fruit snack choices. Taking all of these into consideration, in general, decreasing added sugar content, increasing fiber content, and enhancing overall sensory liking is a direction for product development in the fruit snack category to comply with dietary guidance. Nonetheless, changes to the sensory profile of fruit snacks may not be accepted by consumers who are accustomed to certain sensory characteristics of fruit snacks. Together, product developers and sensory scientists should consider the multisensory properties of the fruit snack and identify if there are sensory properties consumers are willing to compromise on to achieve a healthier snack product.

The current sugar reduction strategies in food products can be classified into four categories. They include using sugar substitutes, integrating multisensory elements, innovating food structure, and decreasing sugar usage gradually over time [66,67]. These approaches require product reformulation, in which sweeteners, texture modifiers, or enhancement of other sensations (aroma, visual stimuli) could be applied to maintain sweet perception while removing sugar from food products. For fruit snack products, using sugar substitutes in product formulation could be an optimal approach to reducing added sugar content, because some natural sweeteners are considered to be low-calorie prebiotic carbohydrates, like oligosaccharide and polysaccharide, which contribute to dietary fiber [68,69]. Therefore, using sugar substitutes can achieve a reduction in added sugar and enhance fiber content at the same time. There have been examples of the use fructo-oligosaccharide (FOS), maltodextrin, tagatose, xylo-oligosaccharide (XOS), and galacto-oligosaccharide (GOS) to achieve added sugar reduction and fiber content enhancement in food products [70,71,72,73,74]. However, the sensory profile of food products should be fully considered in product reformulation by incorporating commercial or novel oligosaccharides, as its sweet intensity is lower than sucrose, and it may have other sensory notes accompanying sweetness, such as bitter, sour, and metallic [69,75]. While these are common strategies which are already applied across food products to achieve sugar reduction, these approaches are not often easily executed due to the product’s composition or processing, or they may not be feasible due to cost. Moreover, these changes have the potential to impact product quality, shelf-life, and consumer acceptance.

### 4.4. Limitations

In this study, the NRF6.4 model utilizes ten nutrients, Protein, Dietary Fiber, Potassium, Vitamin D, Calcium, Iron, Saturated Fat, Cholesterol, Added Sugar, and Sodium, to assess fruit snack nutrient density. The selection of nutrients used in the NRF model could change the outcome of the study [17,21]. For example, fruit contains naturally occurring sugar, which varies depending on the fruit type. Therefore, if total sugar was used instead of added sugar in the NRF model, it is anticipated that the NRF scores would be reduced for products containing more sugar from the base fruit in the product, which is not included in the calculation of added sugar. However, the ten nutrients used in this study were selected based on the updated list of nutrients required to be included on the label by the FDA. This study collected fruit snack product information from the Mintel GNPD platform. Mintel specialists collect and report product information from local stores and websites, which are at risk for process errors. This includes duplicative products and missing information. Prior to analyzing the data, additional searches were performed to obtain missing product information. Interestingly, the number of fruit snacks launched in 2022 was less than in previous years. Nevertheless, it does not represent a decrease in the popularity of fruit snacks but rather a delay in data collection and updating on the Mintel GNPD. Therefore, the variety of fruit snacks in the year 2022 may not be accurately described in the present study and is expected to increase in the future.

## 5. Conclusions

When examining the fruit snack category in the United States over the past six years, fruit snack products’ nutrition density, added sugar content, and fiber content vary significantly across the nine product categories. The present study reveals that only dried fruit, fruit puree, and canned fruit with juice are nutrient-dense, high in fiber, and low in added sugar, which can be nutritious fruit snack options and meet current USDA dietary recommendations on low-energy and high-nutrition snack profiles. This demonstrates that different processing methods can achieve nutrient-dense snack products that are nutritious, according to dietary recommendations. The NRF6.4 score, added sugar, and fiber content are used collectively to inform us on areas of opportunities for improvement, providing a more comprehensive assessment of the nutrient profile. It is possible that the nutrient density calculated using the NRF model could vary based on the nutrients selected, yet based on the nutrients selected in the present study, it is recommended that reducing added sugar content and increasing the fiber content of fruit snacks is one way for consumers to meet the current dietary recommendations for snacks. As fruit is often considered a healthy snack, more work is needed to understand consumers understanding of the nutritional value and healthfulness of fruit snacks and snacking behavior. For example, serving sizes and recommended servings may not resemble consumer consumption behavior. Additionally, modifications that improve the nutritional profile (e.g., higher fiber or lower sugar) may have a negative influence on quality and sensory attributes. Continued research on the nutrition profile of fruit snack products and consumer behavior can help to inform policies, such as recommended snacking consumption and product labeling (i.e., fruit serving equivalents). As consuming fruit snacks is growing in popularity, nutrition profiling is crucial for educating and communicating with consumers about smart food choices and updating dietary guidelines or policies to maximize people’s health and wellness.

## Figures and Tables

**Table 1:** Description of different product categories of fruit snacks.

Fruit Snack Product Category	Description
Canned fruit	Fruit or cut fruit preserved in concentrations other than fruit juice. This could be syrup, artificial/natural sweetener solution, or salt water. Product category can be inferred from product name or description
Canned fruit with juice	Fruit or cut fruit preserved in fruit juice; product category is usually indicated in the product name or description
Dried fruit	Dehydrated or dried fruit; do not contain added sugar, artificial colors and additional flavors; shelf stable
Dried fruit, flavored	Dehydrated or dry fruit; flavored by sugar, salt, fruit juice concentration, or have a flavor coating other than chocolate, like, yogurt, peanut butter, etc.; shelf stable
Formed fruit	Fruit ingredients are the main components, nearly all the ingredients are fruit ingredients; deveral commercially popular products, like fruit bar, fruit leather, fruit strip, fruit roll, fruit twists and fruit jerky, are included in this product category
Fruit chips	The word “chips” indicated from the product name; real fruit in the chip shape format
Fruit-based bar	Have a real fruit component; the product ingredient list is complex, but a fruit-based ingredient is ranked in the first five places, which includes fruit as well as other ingredients (vegetable, cereal, protein, nuts, etc.)
Fruit puree	Fluid food product in pouch; fruit and fruit puree are the main components; product category could be indicated by the product image and package type
Fruit-flavored snack	Fruit snacks that have fruit juice/puree concentrate as well as added sugar components (cane sugar, corn syrup, etc.). Fruit ingredients sit in the top 3 positions; most of products have “have real fruit juice, fruit flavor” in the product description

**Table 2:** Changes on the Daily Value for nutrients in NRF6.4 model based on a 2000 kcal diet.

Nutrient	Daily Value	Change in Requirements
Saturated Fat	20 g	No change
Cholesterol	300 mg	No change
Sodium	2300 mg	Decrease (100 mg)
Dietary Fiber	28 g	Increase (3 g)
Added Sugar	50 g	New
Potassium	4700 mg	Increase (2200 mg)
Vitamin D	20 mcg	No change *
Iron	18 mg	No change
Calcium	1300 mg	Increase (300 mg)

* There was no change in the requirement, but there was a change in the units (from international units (IU) to micrograms (mcg)).

**Table 3:** Number and percentage (% in the cell, percentage accumulates 100%) of new fruit snacks launched in the U.S. Market in each specific product category from 2017 to 2022.

Fruit Snacks Category	2017	2018	2019	2020	2021	2022	Total
Canned fruit	58 (26%)	57 (26%)	27 (12%)	27 (12%)	28 (13%)	25 (11%)	222
Canned fruit with juice	20 (12%)	30 (19%)	22 (14%)	26 (16%)	32 (20%)	32 (20%)	162
Dried fruit	87 (16%)	76 (14%)	103 (19%)	78 (14%)	128 (23%)	73 (13%)	545
Dried flavored fruit	68 (21%)	61 (18%)	49 (15%)	33 (10%)	61 (18%)	58 (18%)	330
Formed fruit	29 (19%)	26 (17%)	32 (21%)	33 (22%)	17 (11%)	16 (10%)	153
Fruit-based bar	72 (23%)	61 (19%)	74 (23%)	52 (16%)	25 (8%)	33 (10%)	317
Fruit chips	45 (24%)	30 (16%)	49 (26%)	22 (12%)	26 (14%)	16 (9%)	188
Fruit-flavored snack	51 (20%)	42 (16%)	44 (17%)	40 (15%)	41 (16%)	43 (16%)	261
Fruit puree	48 (21%)	34 (15%)	32 (14%)	44 (19%)	29 (13%)	40 (18%)	227
Total number of products	478	417	432	355	387	336	2405

**Table 4:** Mean and standard deviation (±SD) of *NR* (sub scores of nutrients to encourage), *LIM* (sub scores of nutrients to limit), Energy Density, and NRF6.4, median NRF6.4, and IQR NRF6.4 for each fruit snack product category based on 100 g.

	N	NR	LIM	Energy Density	NRF6.4
		Mean (±SD)	Mean (±SD)	Mean (±SD)	Mean (±SD)
Canned fruit	114	15.3 (14.7)	25.3 (16.6)	72.7 (44.8) ^a^	−10 (17.4) ^d^
Canned fruit with juice	110	14.4 (5.9)	0.5 (2.8)	55.9 (7.6) ^a^	13.9 (6.8) ^b^
Dried fruit	377	22.4 (10.7)	1.5 (6.1)	323.8 (62.4) ^b^	20.9 (12.5) ^a^
Dried flavored fruit	186	11.3 (8.4)	24.4 (11.7)	356.8 (62.6) ^c^	−13.1 (15.8) ^d^
Formed fruit	96	17.6 (12.1)	6.8 (8.5)	314.4 (63.5) ^b^	10.8 (16.7) ^bc^
Fruit-based bar	209	20.7 (9.1)	15 (9.2)	403.3 (58.7) ^d^	5.1 (12.5) ^c^
Fruit chips	114	11.1 (5)	18.4 (14.1)	509.5 (76.2) ^e^	−7.2 (14.8) ^d^
Fruit-flavored snack	132	2.4 (3.2)	31.8 (6.3)	326.2 (45.9) ^b^	−29.5 (6.4) ^e^
Fruit puree	159	18.2 (11.8)	5 (8.2)	68.5 (19.7) ^a^	13.1 (16.6) ^b^

Letters within the column denote the significant difference from each other by Dunn post hoc test (*p* < 0.05).

**Table 5:** Mean and standard deviation (±SD) for energy density, added sugar, and fiber content of fruit snacks by product category.

		Energy Density		Added Sugar		Fiber	
Fruit Snacks Category	N	per RACC	per 100 g	per RACC	per 100 g	per RACC	per 100 g
		Mean (SD)	Mean (SD)	Mean (SD)	Mean (SD)	Mean (SD)	Mean (SD)
Canned fruit	115	101.8 (62.8)	72.7 (44.8) ^a^	13.1 (13) ^d^	9.4 (9.3) ^d^	1.5 (1.1) ^c^	1.1 (0.8) ^cd^
Canned fruit with juice	110	78.3 (10.7)	55.9 (7.6) ^a^	0.2 (1.1) ^a^	0.1 (0.8) ^a^	1.2 (0.5) ^c^	0.8 (0.4) ^de^
Dried fruit	377	129.5 (25)	323.8 (62.4) ^b^	0.2 (1.6) ^a^	0.4 (4) ^a^	3.5 (2.1) ^a^	8.8 (5.3) ^a^
Dried flavored fruit	186	142.7 (25)	356.8 (62.6) ^c^	13.8 (8.6) ^d^	34.4 (21.4) ^e^	2.2 (2.2) ^c^	5.5 (5.5) ^b^
Formed fruit	96	94.3 (19.1)	314.4 (63.5) ^b^	3.2 (4.4) ^b^	10.6 (14.6) ^c^	2.3 (0.9) ^bc^	7.8 (3.1) ^a^
Fruit-based bar	209	161.3 (23.5)	403.3 (58.7) ^d^	6.1 (5.2) ^c^	15.3 (13.1) ^d^	3 (1.6) ^ab^	7.6 (4) ^a^
Fruit chips	114	152.9 (22.9)	509.5 (76.2) ^e^	2 (3.3) ^b^	6.5 (11.1) ^bc^	2.2 (1.4) ^c^	7.4 (4.6) ^a^
Fruit-flavored snack	132	97.9 (13.8)	326.2 (45.9) ^b^	14.6 (3.5) ^e^	48.6 (11.7) ^f^	0.1 (0.6) ^d^	0.4 (1.9) ^e^
Fruit puree	159	85.6 (24.6)	68.5 (19.7) ^b^	2.2 (4.8) ^b^	1.8 (3.9) ^b^	2.3 (1.5) ^c^	1.9 (1.2) ^c^

Letters within each column denote significant differences from each other by Dunn post hoc test (*p* < 0.05).

## Data Availability

Data will be provided upon request.
